# TGF-β-Dependent Growth Arrest and Cell Migration in Benign and Malignant Breast Epithelial Cells Are Antagonistically Controlled by Rac1 and Rac1b

**DOI:** 10.3390/ijms18071574

**Published:** 2017-07-20

**Authors:** Catharina Melzer, Juliane von der Ohe, Ralf Hass, Hendrik Ungefroren

**Affiliations:** 1 Biochemistry and Tumor Biology Lab, Department of Obstetrics and Gynecology, Hannover Medical School, Hannover 30625, Germany; melzer.catharina@mh-hannover.de (C.M.); ohe.juliane.von.der@mh-hannover.de (J.v.d.O.); 2 Department of General and Thoracic Surgery, Universitatsklinikum Schleswig-Holstein, Campus Kiel, Kiel 24105, Germany

**Keywords:** breast cancer, HMEC, cell cycle arrest, senescence, transforming growth factor-β

## Abstract

Despite improvements in diagnosis and treatment, breast cancer is still the most common cancer type among non-smoking females. TGF-β can inhibit breast cancer development by inducing cell cycle arrest in both, cancer cells and, as part of a senescence program in normal human mammary epithelial cells (HMEC). Moreover, TGF-β also drives cell migration and invasion, in part through the small GTPases Rac1 and Rac1b. Depletion of Rac1b or Rac1 and Rac1b in MDA-MB-231 or MDA-MB-435s breast cancer cells by RNA interference enhanced or suppressed, respectively, TGF-β1-induced migration/invasion. Rac1b depletion in MDA-MB-231 cells also increased TGF-β-induced p21^WAF1^ expression and ERK1/2 phosphorylation. Senescent HMEC (P15/P16), when compared to their non-senescent counterparts (P11/P12), presented with dramatically increased migratory activity. These effects were paralleled by elevated expression of genes associated with TGF-β signaling and metastasis, downregulated Rac1b, and upregulated Rac1. Our data suggest that acquisition of a motile phenotype in HMEC resulted from enhanced autocrine TGF-β signaling, invasion/metastasis-associated gene expression, and a shift in the ratio of antimigratory Rac1b to promigratory Rac1. We conclude that although enhanced TGF-β signaling is considered antioncogenic in HMEC by suppressing oncogene-induced transformation, this occurs at the expense of a higher migration and invasion potential.

## 1. Introduction

Breast cancer is one of the most frequently diagnosed neoplasms in women and the leading cause of cancer death among non-smoking females [1]. Treatment of this malignancy is still a challenge to clinicians because of our incomplete knowledge of the underlying molecular alterations that drive breast cancer development and metastasis. Gene expression profiling studies have revealed different phenotypes of breast carcinomas. Among these, triple-negative breast carcinomas (estrogen receptor negative, progesterone receptor negative, Her2/Neu negative), which often have a poor prognosis, display a basal-like gene expression profile similar to that of human mammary epithelial cells (HMEC). HMEC are therefore considered an appropriate model for the study of basal-like breast cancer.

Overexpression of TGF-β and high autocrine TGF-β signaling in tumor cells has been implicated in promoting immune suppression, tumor angiogenesis, tumor cell migration, invasion, and metastasis in many cancers, including carcinoma of the breast [2]. Various components of the TGF-β signaling pathway have been shown to be mutated or altered in expression in certain types of carcinomas, such as *DPC4*, encoding the intracellular signal transducer Smad4, in pancreatic cancer. Although mutations in *DPC4* also occur in a subset of human breast cancer, mutational inactivation of TGF-β receptors or Smad proteins is not as common as in pancreatic or colon cancer [3]. Rather, many breast cancer cells and tissues show gene signature expression profiles resembling RAS activation [4]. Overexpressed or hyperactivated RAS and its downstream effectors, RAF and MEK, all can induce cell cycle arrest and premature senescence (termed oncogene-induced senescence, OIS, in the case of mutated RAS). For instance, H-Ras-V12 induces OIS in HMEC in vitro by activating p16^INK4a^/Rb and p53 pathways [5]. In fact, senescence-like phenotypes have been reported in early stages of breast tumors [6,7]. TGF-β receptor signaling has been shown to be protective against oncogene-induced transformation and malignant progression in HMEC. Bypassing OIS e.g., by attenuation of TGF-β signaling in mouse keratinocytes was shown to overcome H-Ras-V12–induced premature senescence [8] and to accelerate tumorigenesis. More recently, blockade of TGF-β signaling by expression of dominant-negative TGF-β receptor type II (TβRII) in telomerase-immortalized HMEC ectopically expressing H-Ras-V12 suppressed H-Ras-V12-induced senescence-associated growth arrest via loss of p21^WAF1^ expression and rendered HMEC highly tumorigenic and metastatic in vivo [9].

Even without overt oncogene activation, HMEC in vitro undergo aging between passage 11 (P11) and passage 16 (P16) to reach a senescent phenotype and cell cycle arrest at P16 [10,11]. Different mechanisms can promote senescence including STASIS (stress- or aberrant signaling-induced senescence) mediated by increased oxidative stress and DNA damage or intracellular replicative senescence triggered by the progressive erosion and eventual dysfunction of telomeres during the proliferative cell cycle [12]. Extensive study of cultured HMEC has identified two senescence barriers. One involves induction of the cyclin-dependent kinase inhibitor p16 before attaining critically short telomeres. This STASIS barrier can be overcome by inhibiting p16, allowing continued proliferation, which results in agonescence, a proliferative barrier mediated by telomere depletion [13]. However, senescent HMEC may have the potential for malignant transformation and initiation of breast cancer development due to enhanced manganese-dependent superoxide dismutase (MnSOD) activity and ROS production [14]. In the agonescent state, some HMEC can accumulate chromosome abnormalities resulting in the maintenance of a viable population with renewed proliferative potential [15].

HMEC exhibit autocrine TGF-β signaling which is considered an integral part of cellular antitransformation network by suppressing the expression of genes that mediate oncogene e.g., Ras-induced transformation. In both HMEC and in breast carcinoma cells TGF-β signaling inhibits breast carcinogenesis also by inducing cell cycle arrest, e.g., via induction of p21^WAF1^. In the carcinoma cells, TGF-β cooperates with Ras-Raf-MEK-ERK signaling to induce p21^WAF1^ [9] but also to promote EMT and cell motility. In line with this, disruption of TGF-β signaling (by ectopic expression of dominant-negative TβRII or deletion of TβRII) in HMEC has been found to increase cell proliferation, with no evidence of tumorigenesis in mammary gland, pancreas, and epidermis in mice [16,17]. However, whether the enhanced autocrine TGF-β signaling in senescent growth arrested HMEC also leads to an increase in cell motility in vitro has not been tested so far.

Rac1 and its splice variant, Rac1b, are members of Rho family of small GTPases that are abundantly expressed in breast and pancreatic carcinoma [18]. Rac1b expression in ductal carcinoma cells of pancreatic cancer patients has been shown to be beneficial with respect to overall survival [19]. Rac1 is a crucial component of the ROS producing NADPH oxidases and both Rac1 and Rac1b have been implicated in ROS production and ROS-dependent signaling [20,21]. Rac1b, but not Rac1, has been suggested to inhibit the B-RAF-induced senescence phenotype and upregulation of the cell-cycle inhibitors p14^ARF^, p15^INK4B^ and p21^WAF1^ in a ROS-dependent manner [22] and, therefore, may represent a crucial factor linking TGF-β-induced cell motility and senescence-like growth arrest at the molecular level. Based upon the observations that (i) TGF-β induces cell cycle arrest in breast carcinoma cells that have retained responsivity to this growth factor and (ii) senescence-associated cell cycle arrest was associated with enhanced TGF-β signaling, we asked whether TGF-β1-arrested malignant breast epithelial cells and senescent HMEC are more motile and, if so, whether the migratory phenotype is controlled by Rac1 and/or Rac1b. To test these ideas, we compared the TGF-β1-arrested growth states of breast carcinoma cells and their presumed untransformed progenitors, the HMEC, and addressed the following issues: (i) Do Rac1 and Rac1b alter TGF-β1-induced cell migration and cell cycle arrest in breast carcinoma cells? (ii) Is acquisition of a senescence-associated growth arrest phenotype in HMEC associated with alterations in Rac1 and/or Rac1b expression? (iii) Is a senescent growth arrested phenotype associated with increased migratory activity in HMEC and are these effects associated with altered expression of invasion/metastasis-associated genes? The results from this study may provide useful information in devising prevention and treatment strategies for more selective and specific targeting of TGF-β in breast cancer.

## 2. Results

### 2.1. Depletion of Rac1 Suppresses While Rac1b Enhances Basal and TGF-β1-Induced Migration of Breast Cancer Cells

**Figure 1:**
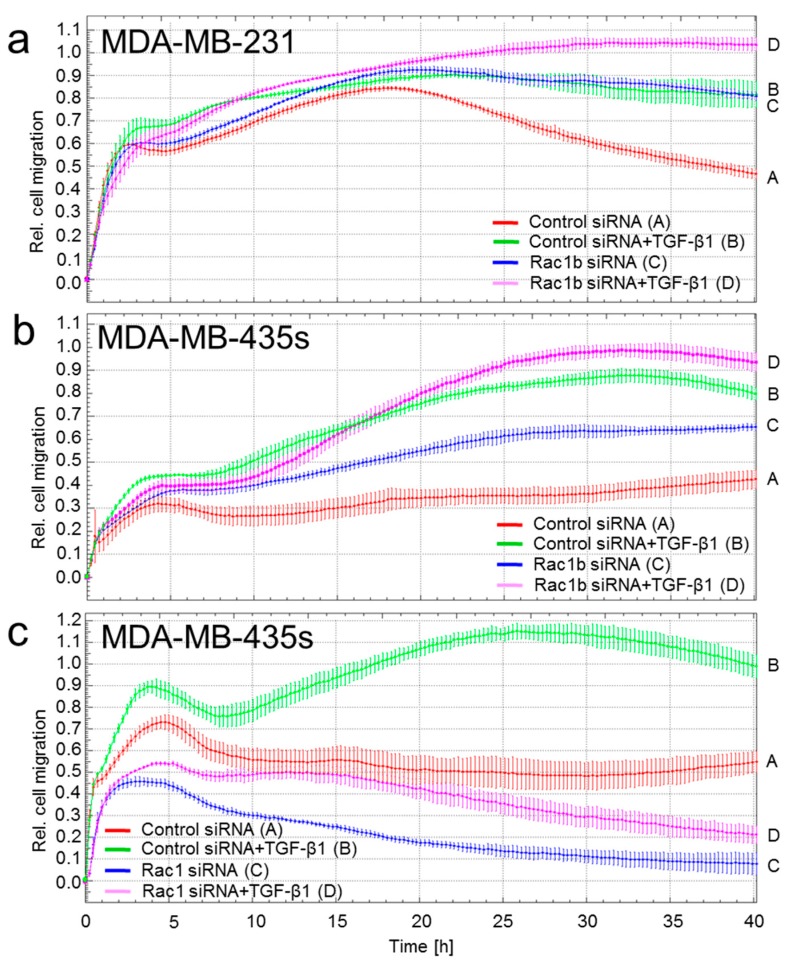
Effect of Rac1b or Rac1 + Rac1b depletion on TGF-β1-mediated cell migration in MDA-MB-231 and MDA-MB-435s cells. (**a**) MDA-MB-231 cells were transiently transfected twice at two consecutive days with 50 nM of either control siRNA or Rac1b siRNA. Forty-eight hour later, cells were subjected to chemokinesis assay in the presence or absence of recombinant human (rh) TGF-β1 (5 ng/mL). Differences are significant between control siRNA (red curve, tracing A) and Rac1b siRNA (blue curve, tracing C) and between control siRNA + TGF-β1 (green curve, tracing B) and Rac1b siRNA + TGF-β1 (magenta curve, tracing D) at 24:00 and all later time points; (**b**) as in (A) except that MDA-MB-435s cells were used. Differences are significant between control siRNA (red curve, tracing A) and Rac1b siRNA (blue curve, tracing C) at 15:00 and all later time points; (**c**) as in (A) except that the Rac1b siRNA was replaced by an siRNA that recognizes both Rac1 and Rac1b. Differences are significant between control siRNA (red curve, tracing A) and Rac1 siRNA (blue curve, tracing C) and between control siRNA + TGF-β1 (green curve, tracing B) and Rac1 siRNA + TGF-β1 (magenta curve, tracing D) at the 5:00 and all later time points. In each panel, three experiments were performed with very similar results of which one is shown.

Previous data with pancreatic cancer cells have shown that Rac1b acts as an endogenous inhibitor of TGF-β signaling by interfering with Smad2/3 activation [19]. We therefore speculated that Rac1b antagonizes TGF-β1-induced cell motility in breast carcinoma cells and, consequently, that alterations in its expression impact the invasive behavior of mammary epithelial cancer cells. To provide functional proof of this hypothesis, we performed realtime cell migration assays with two triple-negative carcinoma cell lines (MDA-MB-435s, MDA-MB-231) that express readily detectable levels of both Rac1 and Rac1b. MDA-MB-435s and MDA-MB-231 cells were selectively depleted of Rac1b using a siRNA that targets exon 3b unique to Rac1b [19]. As shown in Figure 1, depletion of Rac1b enhanced TGF-β1-induced migration in both MDA-MB-231 (Figure 1a, magenta vs. green curve) and MDA-MB-435s (Figure 1b, magenta vs. green curve) cells. Rac1b depletion in both cell lines also enhanced migration of non-stimulated control cells (blue vs. red curve), which may represent migration driven by autocrine TGF-β. When MDA-MB-435s cells were depleted of Rac1 and Rac1b by transient transfection with an siRNA targeting, both proteins they exhibited strongly reduced migration under both non-stimulated and TGF-β1 stimulated conditions (Figure 1c). These data clearly show that Rac1b acts as an endogenous inhibitor of chemokinesis in malignant mammary epithelial cells.

### 2.2. Depletion of Rac1b in MDA-MB-231 Cells Increases TGF-β-Induced p21^WAF1^ Expression and ERK1/2 Phosphorylation

**Figure 2:**
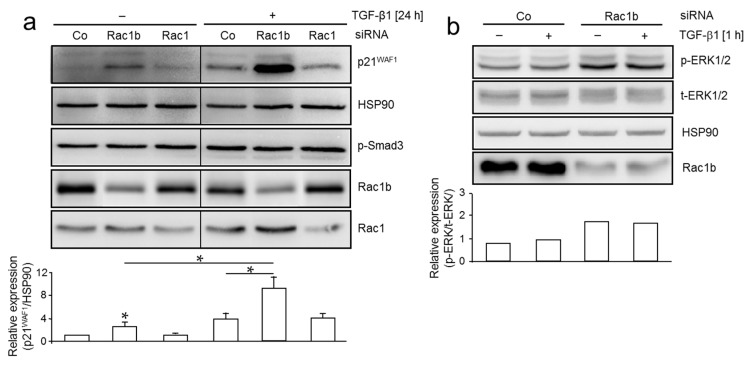
Depletion of Rac1b in MDA-MB-231 cells increases TGF-β-induced p21^WAF1^ expression and ERK1/2 phosphorylation. (**a**) MDA-MB-231 cells were transiently transfected twice at two consecutive days with 50 nM of either a control siRNA (Co), a Rac1b-specific siRNA, or a Rac1-specific siRNA not cross-reactive with Rac1b. Twenty-four h after the second transfection, cells were starved (0.5% FBS) overnight and subsequently treated in the same medium with TGF-β1 for 24 h followed by immunoblot analysis of p21^WAF1^, phospho-Smad3 (p-Smad3), and HSP90 as loading control. Successful depletion of Rac1b and Rac1 protein was verified with a Rac1b-specific antibody and a Rac1 antibody, respectively. Onerepresentative blot is shown withall bands being from the same blot. The vertical lines between lanes of control and TGF-β-treated samples indicate removal of irrelevant lanes. The graph below the blot depicts results from a densitometric analysis using NIH Image J of underexposed replicates from three independent experiments (mean ± SD, *n* = 3). * *p* < 0.05; (**b**) MDA-MB-231 cells were transiently transfected twice at two consecutive days without transfection reagent alone (-), or with 50 nM of either a control siRNA (Co), or a Rac1b-specific siRNA and further processed as described in (**a**) except that treatment with TGF-β1 was for 1 h. Immunoblots were incubated with antibodies to phospho-ERK1/2 (p-ERK1/2), total-ERK1/2 (t-ERK1/2), and Rac1b and HSP90 as controls. The immunoblot shown is representative of three independent experiments.

TGF-β1 has been shown to induce growth inhibition in breast cancer cells via upregulation of p21^WAF1^. Moreover, ectopic expression of Rac1b inhibits B-RAF-induced senescence and cell cycle arrest in colonic cells and downregulates p21^WAF1^ [22]. To analyze the consequences of cellular Rac1b depletion for TGF-β1-induced growth inhibition, we inhibited Rac1b by RNAi in MDA-MB-231 cells and monitored expression of p21^WAF1^. Its expression was induced by TGF-β1 in control cells in agreement with cell cycle arrest/senescence (Figure 2a). Notably, cells that were depleted of Rac1b presented with strongly increased basal and TGF-β1-induced protein levels of p21^WAF1^ relative to cells that received control siRNA or a Rac1-specific siRNA (Figure 2a). Regardless of different forms of treatment, the cells displayed readily detectable levels of phospho-Smad3 suggesting activation of the Smad pathway by autocrine TGF-β (see above). Since Rac1b has been shown to inhibit growth arrest induced by B-RAF, the upstream activator of MEK-ERK signaling, which in turn is crucial for both basal and TGF-β1-induced cell migration and p21^WAF1^ expression, we asked whether Rac1b would affect ERK activation in MDA-MB-231 cells. Intriguingly, MDA-MB-231 cells depleted of Rac1b presented with increased levels of phospho-ERK1/2 (Figure 2b). From these data, we conclude that Rac1b is a potent endogenous inhibitor of both TGF-β1-induced p21^WAF1^ expression and ERK activation in human breast carcinoma cells.

### 2.3. Differential Expression of Metastasis-Associated Genes in HMEC of Low and High Passage Number

**Figure 3:**
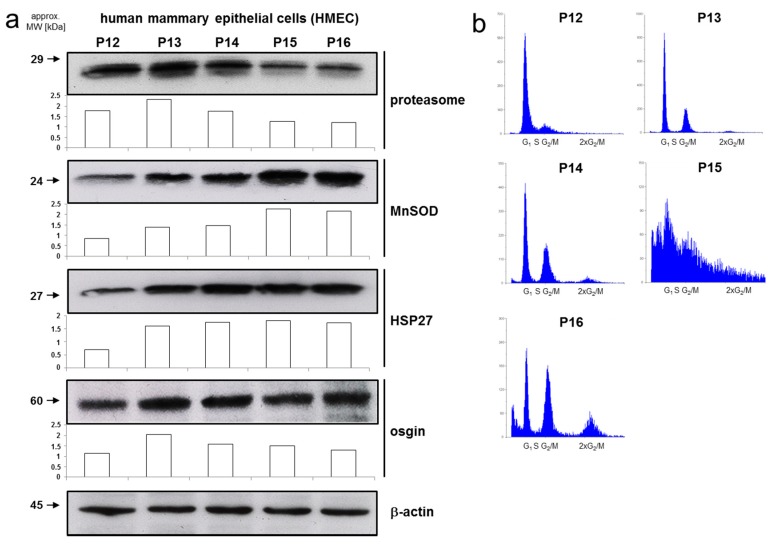
Characterization of the senescent phenotype of HMEC between P12 and P16. (**a**) HMEC of different passage numbers were lysed and subjected to immunoblot analysis of the indicated proteins. Detection of β-actin served as loading control. Intensities were quantified by NIH Image J with corresponding ^®^-actin protein bands as a reference; (**b**) Cell cycle distribution in HMEC of different passage numbers.

We have previously shown that HMEC become senescent in in vitro culture between P12 and P16, while HMEC up to P11 were designated young, non-senescent cells [10,11]. Cellular aging in these cells is associated with morphological alterations such as an increased cell size [23]. Functional alterations after P12 are evident from (i) reduced expression of the proteasome (Figure 3a), eventually resulting in accumulation of damaged proteins due to lower poly-ubiquitinylation or poly-ADP-ribosylation activity, (ii) induction of MnSOD leading to enhanced production of superoxide (Figure 3a), as well as of heat shock protein 27 (HSP27) and osgin (oxidative stress induced growth inhibitor 1) cellular responses to enhanced oxidative stress and damage (Figure 3a). The cell cycle distributions among the different HMEC populations between P11 and P16 revealed decreasing levels of G0/G1 and S phase cells (in P16) with increasing culture time while cell cycle populations in G2/M significantly increased (Figure 3b). Another population in the range of 2 × G2/M continuously accumulated between P11 and P16 indicating aberrant mitosis by DNA doubling without subsequent cell division (Figure 3b). These findings suggested a strongly reduced proliferative capacity at P16 which had been confirmed earlier by cell number and viability measurements [10]. The senescent phenotype of P15/P16 HMEC has been validated in previous work by the demonstration of markers of (senescence-associated) cell cycle arrest including p15^INK4B^ [10,11].

To validate whether high-passage HMEC differ from low-passage cells in the expression of potentially harmful genes (e.g., those governing invasion and metastasis and to assess endogenous activity of TGF-β signaling in these cells), we subjected pooled RNA fractions of low-passage HMEC versus high-passage HMEC to qPCR analysis with the RT^2^ Profiler™ PCR Array “Human Tumor Metastasis.” Following normalization with the housekeeping genes, we identified genes that are upregulated in senescent and migration-active cells (∆C_t_ (threshold cycle) ≥ 1.5), e.g., *APC*, *CD44*, *CDKN2A*, *COL4A2*, *CXCR2*, *CXCR4*, *EPHB2*, *FXYD5*, *KRAS*, *MET*, *MMP2*, *PLAUR*, *MGAT5*, *SMAD2* and *SMAD4* (Table 1). Notably, only two genes (*KISS1* and *KISS1R*) were found to be downregulated (Table 1). The majority of these genes is involved in various aspects of TGF-β biology, e.g., signal transducers (*SMAD2*, *SMAD4*), target genes of TGF-β (*CD44* [24,25], *CDKN2A* encoding p16^INK4A^ [26], *COL4A2* [27], *CXCR4* [28,29], *MMP2* [30], *PLAUR* encoding urokinase-type plasminogen-activator receptor (PAR [31,32]), or are regulators involved in TβRII endocytotic regulation (mannosyl (α-1,6-)-glycoprotein β-1,6-*N*-acetyl-glucosaminyltransferase V, MGAT5 [33]). These data confirm earlier observation of enhanced autocrine TGF-β signaling in senescent HMEC [9].

### 2.4. A Senescent Phenotype of HMEC was Associated with an Increase in Rac1 Expression

**Figure 4:**
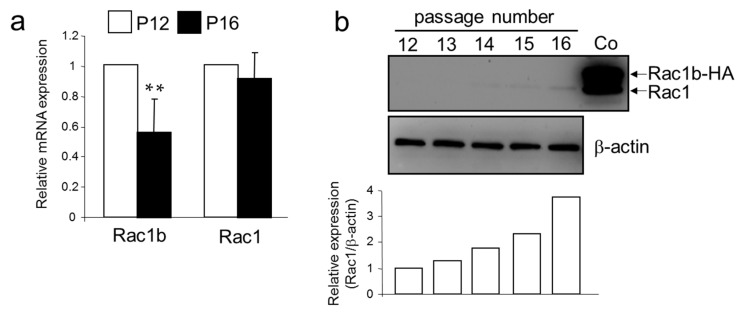
Rac1b and Rac1 expression in HMEC of different passage numbers. (**a**) qPCR-based measurement of Rac1b and Rac1 expression in P12 and P16 HMEC. Data are the mean ± SD of three experiments (*n* = 3) and those from P16 HMEC are expressed relative to those in P12 HMEC set arbitrarily to 1.0; (**b**) Immunoblot-based measurement of Rac1 in HMEC of different passage numbers as indicated. As control (Co), a lysate from the pancreatic cancer cell line Panc1 ectopically expressing Rac1b-HA was used. A densitometric analysis of underexposed replicas is shown below the blots. Data are representative of three experiments.

Results from above suggest that senescent HMEC have higher activity of endogenous TGF-β signaling and a more invasive gene signature. To test the possibility that this is caused by changes in Rac1b and/or Rac1 expression, we determined endogenous levels of Rac1 and Rac1b in young and aging HMEC. Interestingly, qPCR analysis revealed that expression of Rac1b in senescent HMEC (P12) was lower than in young HMEC (P12) (Figure 4a). However, in immunoblot analysis, Rac1b was not detectable, while we realized an almost four-fold increase in Rac1 protein abundance (Figure 4b) between passages 12 and 16. Hence, HMEC appear to upregulate Rac1 and downregulate Rac1b expression via different mechanisms, thus further increasing the ratio of Rac1b to Rac1 expression.

### 2.5. A Senescent Phenotype is Associated with Increased Migratory Activity in HMEC

**Figure 5:**
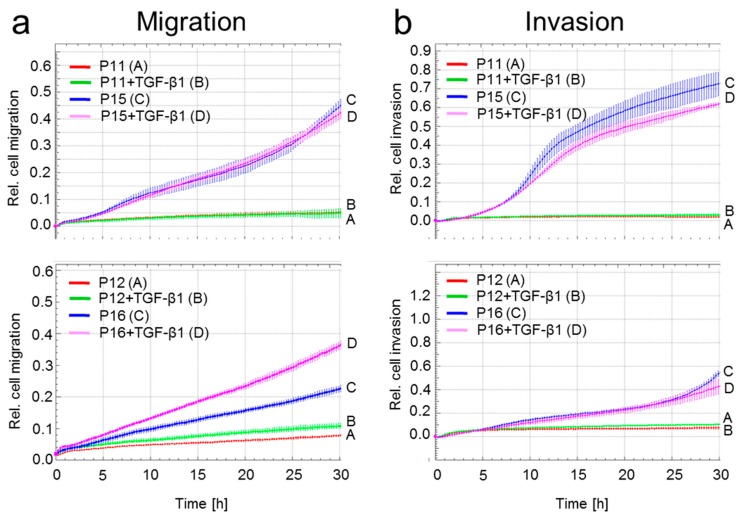
Effect of passage number on basal and TGF-β1-mediated cell migration in HMEC. (**a**) P11 and P15 (upper graph) and P12 and P16 (lower graph) HMEC were subjected to migration assay in the presence or absence of rhTGF-β1 (10 ng/mL). For P11 and P15 (upper graph) differences are significant between P11 HMEC (red curve, tracing A) and P15 HMEC (blue curve, tracing C) and between P11 HMEC + TGF-β1 (tracing B) and P15 HMEC + TGF-β1 (tracings C and D) at 10:00 and all later time points. For P12 and P16 (lower graph) differences are significant between P12 HMEC (red curve, tracing A) and P16 HMEC (blue curve, tracing C) and between P12 HMEC + TGF-β1 (green curve, tracing B) and P16 HMEC + TGF-β1 (magenta curve, tracing D) at 8:00 and all later time points; (**b**) P11 and P15 (upper graph) and P12 and P16 (lower graph) HMEC were subjected to invasion assay with matrigel as barrier in the presence or absence of rhTGF-β1 (10 ng/mL). For P11 and P15 (upper graph) differences are significant between P11 HMEC (red curve, tracing A) and P15 HMEC (blue curve, tracing C) and between P11 HMEC + TGF-β1 (green curve, tracing B) and P15 HMEC + TGF-β1 (magenta curve, tracing D) at 8:00 and all later time points. For P12 and P16 (lower graph) differences are significant between P12 HMEC (red curve, tracing A) and P16 HMEC (blue curve, tracing C) and between P12 HMEC + TGF-β1 (green curve, tracing B) and P16 HMEC + TGF-β1 (magenta curve, tracing D) at 20:00 and all later time points.

Microarray analysis indicated higher endogenous TGF-β signaling, higher expression of Ki-RAS and upregulation of invasion-associated genes in P15/P16 HMEC. Moreover, senescent growth-arrested HMEC had low expression of Rac1b but high expression of the promigratory Rac1. In an earlier study, we observed that young (P11 and P12) HMEC did not show any chemokinetic activity in xCELLigence assays [34]. These observations led us to assume that HMEC with a senescent phenotype could be migration-active. Intriguingly, we observed a dramatically increased propensity of the senescent HMEC (P15/P16) over young HMEC (P11/P12) for both cell migration (Figure 5a) and cell invasion through a Matrigel layer (Figure 5b). The addition of exogenous rhTGF-β induced only little or no additional migratory activity (Figure 5a,b), which may indicate already high endogenous signaling. These data suggest that the senescent growth-arrested phenotype of HMEC was associated with functional alterations in genes that control cell migration and invasion and that autocrine TGF-β signaling is involved in driving this migratory activity.

## 3. Discussion

The role of TGF-β in the development of breast cancer and other cancer types is complex. While TGF-β signaling suppresses the growth of normal epithelial cells and some cancer cell lines, it is often required for the maintenance of a transformed phenotype by promoting cell invasion and metastasis. The response of normal, hyperplastic, and transformed epithelial cells may be explained by Smad-dependent and independent pathways activated by TGF-β which ultimately determine whether cells arrest, continue to divide, or undergo EMT. In addition, TGF-β can induce growth arrest/premature senescence in response to ionizing radiation or oncogene activation [35]. In the breast carcinoma cell lines MDA-MB-231 and MDA-MB-435s, treatment with rhTGF-β1 induced growth arrest via p21^WAF1^ expression (see Figure 2) in agreement with results from an earlier study [36], and enhanced the cells’ propensity for random cell migration [35].

The cells’ response to exogenous and autocrine TGF-β can be suppressed by expressing a dominant-negative TβRII, use of a TGF-β type I receptor inhibitor, or ectopic expression of MYC (a well-known suppressor of TGF-β signaling) which might prevent RAS-mediated OIS, and together with loss of p16 and p53 function, eventually permits the expansion of HMEC with a malignant phenotype. However, cells can also alter intracellular signaling activity autonomously by activating endogenous promoters and inhibitors such as MYC or, as shown here, members of the Rho/Rac family of small GTPases [37]. Specifically, we have identified Rac1b as a negative regulator of the TGF-β1-induced cell cycle inhibitor p21^WAF1^, thereby acting in a promitotic manner by counteracting TGF-β1-induced growth arrest. We further show in two breast cancer cell lines that Rac1 and Rac1b are powerful modulators of TGF-β1-induced random cell migration, whereby Rac1 promotes and Rac1b inhibits random cell migration [19,37]. Thus, Rac1b might function as a dual repressor of TGF-β-induced migration and senescence-dependent cell cycle arrest. This is supported by a recent study showing that ectopic Rac1b expression bypasses B-RAF-induced senescence and senescence-like growth arrest [22]. However, the downstream targets of Rac1 are not known at present. Since RAF is an upstream activator of ERK1/2 and RAS/RAF/MEK/ERK signaling is essential for TGF-β-induced cell migration/invasion in breast epithelial cells [38,39,40], we monitored activation of this pathway in Rac1b-depleted cells. Notably, in MDA-MB-231 cells which display high basal migratory activity and constitutive ERK activation, levels of ERK1/2 were enhanced after Rac1b depletion and this correlated with higher motility in both TGF-β1-stimulated and in non-stimulated Rac1b-depleted cells in the migration and invasion assays (see Figure 1). The Rac1b siRNA-mediated relief from inhibition of ERK signaling might account for Rac1b siRNA-induced increase in TGF-β1 induction of p21^WAF1^ expression since p21^WAF1^ is upregulated by TGF-β/Smad and MEK-ERK signaling in an oxidative stress/ROS-dependent manner [41,42]. If so, senescence-like growth arrest and cell migration in mammary epithelial cells may both be controlled by Rac1b through its inhibitory effect on the RAS-RAF-MEK-ERK pathway (this study) and the TGF-β/Smad pathway [19]. Indeed, TGF-β/Smad pathway mediates IFNγ/TNFα-evoked genotoxicity and cellular senescence via induction of NADPH oxidases (NOX) and suppression of ANT2 [43] and TGF-β can induce upregulation of NOX4 and ROS production.

Cultures of HMEC spontaneously adopt a senescent growth-arrested phenotype after 15-16 passages in vitro as evidenced by upregulation of SA-β-gal and p16^INK4A^, and other features [10,11]. Autocrine TGF-β enhances the senescent phenotype and premature senescence is often associated with classical TGF-β-mediated responses like growth inhibition, upregulation of p16^INK4A^ and p21^WAF1^, and matrix production [11]. This is supported by a study in hPCS-derived endothelial cells showing that suppression of endogenous TGF-β signaling delays cellular senescence and reduces the expression of p15^INK4B^, p16^INK4A^, and p21^WAF1^ [44]. In addition, disruption of autocrine TGF-β signaling in telomerase-immortalized HMEC with ectopic H-Ras-V12 expression suppressed H-Ras-V12-induced senescence-like growth arrest and rendered these cells highly tumorigenic and metastatic in vivo. The mutant RAS potently or acutely initiates the tumor-suppressive OIS in HMEC. However, RAS signaling elevated via overexpression of growth factor receptors or wild-type RAS (see Table 1) is more frequently observed in breast cancer [45] and may have the same effect.

To validate whether high-passage cells have higher TGF-β signaling activity and differ from low-passage cells in the expression of potentially harmful genes e.g., those governing invasion and metastasis, we subjected low and high passage HMEC to microarray analysis. Results confirm that senescent HMEC have enhanced activity of the TGF-β signaling pathway as evidenced by upregulation of Smad2 and Smad4, p16^INK4A^ [26], COL4A2 [27], MMP-2 [30], uPAR [31,32], CD44 (a cancer stem cell, EMT and migration/invasion-associated gene induced by both TGF-β and ionizing radiation in A549 and MDA-MB-231 cells [24,25]), and MGAT5, an enzyme required for the extracellular domain of TβRII to mediate receptor partitioning into membrane rafts and efficient entrance into caveolin-positive endosomes [33]. EphB2 is involved in the development of breast cancer and increased EphB2 protein expression was negatively associated with overall patient survival [46]. Moreover, KISS1 (encoding Kisspeptin-10) and KISS1R/G-protein-coupled receptor 54 (GPR54) were found to be downregulated in senescent/migration-active HMEC, which is interesting since kisspeptin signaling via its receptor KISS1R/GPR54 has been implicated in the suppression of cell migration [47] and metastasis with a variety of cancers [48] and in the inhibition of human umbilical vein endothelial cell (HUVEC) migration, invasion, and tube formation [49]. Kisspeptin-10 also blocks the activation of c-Src/focal adhesion kinase and Rac/Cdc42 signaling pathways in HUVECs [49]. Higher expression of Ki-RAS in P15/P16 HMEC may result in higher activity of MEK-ERK signaling and consequently enhanced TGF-β-dependent cell motility. With respect to CXCR4, dormant/non-proliferative/slow-cycling disseminated tumor cells in bone marrow highly express TGF-β2 to maintain SDF-1-CXCR4 overexpression and inhibition of SDF-1-CXCR4 signaling by down-regulating TGF-β2 reversed the drug resistance of HNSCC cell line HEp3 via reactivation of cell proliferation [29]. The induction of these genes in senescent HMEC as well as the observation that an accumulation of aging HMEC is associated with enhanced matrix production [23], a hallmark response to TGF-β, suggesting the possibility that the TGF-β also drives a migratory phenotype in senescent HMEC. Indeed, this represents an interesting issue that has not been studied before and would require further knowledge about TGF-^®^ signaling in HMEC. As predicted, HMEC in P15/P16 but not in P11/P12 presented with strongly enhanced random migration (see Figure 4). High autocrine TGF-β signaling in HMEC is also suggested by the observation that treatment of HMEC with rhTGF-β did not or only marginally further increase migration.

Given the known roles of Rac1b and Rac1 in the regulation of Smad signaling in pancreatic cells [19,50], we asked whether these proteins are involved in the acquisition of HMEC migratory activity. Interestingly, the high-passage/aging HMEC display lower expression of Rac1b and higher expression of Rac1 and are thus characterized by a low Rac1b:Rac1 ratio. Hence the promigratory effect might be due to a relief from inhibition of Smad signaling [19] and/or MEK-ERK signaling both of which are required for basal and TGF-β-dependent cell motility. It remains to be seen, if genes identified to be differentially regulated in low passage number/non-migration active cells vs. high passage number/migration-active cells (see above) are indeed regulated by Rac1b and/or Rac1. Co-expression of Rac1b in colonocytes counteracts B-RAF-induced senescence, expression of SA-β-gal, p14^ARF^, p15I^NK4B^ and p21^WAF1^ (which are all regulated by TGF-β), and suppression of proliferation marker Ki67 [22], indicating the selection for increased Rac1b expression as one potential mechanism by which tumor cells can escape from B-RAF-induced senescence. However, both (B-RAF-induced) high level and low level Ras signaling cooperates with the abrogation of TGF-β signaling to promote malignant transformation of breast epithelial cells. It is thus conceivable that elevated Rac1b expression allows for an escape from tumor-suppressive senescence and progress to malignant transformation even in the absence of oncogene activation [51]. In this event, therapeutically targeting Rac1b in HMEC for inhibition would be beneficial since it prevents malignant transformation or even activates the senescence programs in the cancer cells. In contrast, in advanced stages of breast cancer strategies to enhance Rac1b expression and/or lowering Rac1 expression, e.g., by diallyl disulfide, a garlic-derived compound that downregulates the Rac1-ROCK1/PAK1-LIMK1-ADF/cofilin signaling pathway and suppresses cell migration and invasion in human colorectal cancer cells [52], could be effective in treating metastatic disease by interfering with proinvasive TGF-β signaling.

## 4. Materials and Methods

### 4.1. Reagents and Antibodies

The following primary antibodies were used: Anti-phospho-ERK1/2 (#4370, Cell Signalling Technology, Frankfurt/Main, Germany), anti-HSP90 (both #sc-7947 and #sc-13119), anti-ERK1/2 (#AF1576) and phospho-Smad3(Ser423/425) (#AB3226), both from R&D Systems, Wiesbaden, Germany anti-Rac1b (#09-271, Merck Millipore, Darmstadt, Germany), anti-Rac1 (#610650), anti-CIP1/WAF1 (#610233) (both from BD Biosciences, Heidelberg, Germany), anti-proteasome (#PW-8195, Biomol, Plymouth Meeting, PA, USA), anti-HSP27 (#SA-348, Biomol), anti-MnSOD (#06-984, Upstate Cell Signaling Solutions, Lake Placid, NY, USA), anti-osgin (#H00029948-B01P, Abnova, Taipei, Taiwan), anti-β-actin (#A1978, Sigma-Aldrich, Deisenhofen, Germany). HRP-linked anti-rabbit (#7074), anti-mouse (#7076) and anti-rat (#7077) secondary antibodies were from Cell Signaling Technology, anti-goat secondary antibody (#ab6741) was from Abcam (Cambridge, UK). The rhTGF-β1 (#300-023) was purchased from ReliaTech (Wolfenbüttel, Germany) and used at a concentration of 5 ng/mL for the breast cancer cell lines and 10 ng/mL for HMEC.

### 4.2. Cell Cultures of Human Mammary Epithelial Cells

Human mammary epithelial cells (HMEC) were commercially obtained from Lonza Walkersville Inc. (Walkersville, MD, USA). Primary epithelial cells from a 50-year-old caucasian female were isolated from mammary tissue. Cells were received as culture passage 6 (P6) (Lot #92796) and were tested negative for HIV-1, mycoplasma, yeast, fungi, bacteria and hepatitis B and C. HMEC were cultured under humidified conditions at 37 °C, 5% CO_2_ in serum-free mammary epithelial basal medium (MEBM) (Lonza, Cologne, Germany). The medium was supplemented with 52 μg/mL of bovine pituary extract, 0.5 μg/mL of hydrocortisone, 5 μg/mL of human recombinant insulin and 10 ng/mL of human recombinant epidermal growth factor and was substituted every three to four days. At subconfluency, HMEC were passaged as described previously [53]. Briefly, cells were washed with HepesBSS buffer (Lonza) and incubated with 0.025%/0.01% trypsin/EDTA (Lonza) for 6 min at 37 °C. Following complete detachment of cells (which was monitored microscopically), trypsin inactivation with trypsin neutralization solution (TNS) (Lonza) and centrifugation (500 g for 6 min), approx. 4500 cells/cm^2^ were transferred into a new culture flask. In the experiments HMEC of passage 12 (P12) were used.

### 4.3. Culture of Established Breast Cancer Cell Lines

The established human breast carcinoma cell lines MDA-MB-231 (HTB-26) and MDA-MB-435s (HTB-129) were purchased from ATCC and routinely maintained in RPMI 1640 (Lonza) supplemented with 10% FBS, 2 mM l-glutamine, 2 mM sodium pyruvate and penicillin/streptomycin. All cells were routinely tested for the absence of mycoplasma contamination.

### 4.4. Transient Transfection of siRNAs

On day 1 and 2 after seeding into 6-, 12-, or 24-well plates (Nunclon™ Delta Surface, Nunc, Roskilde, Denmark) cells were transfected with 50 nM of an siRNA specific for Rac1b or scrambled control [19], siRNA specific for Rac1 (5′-UAAGAACACAUCUGUUUGCTT-3′), or an siRNA targeting both Rac1 and Rac1b (ON-TARGETplus SMARTpool, a mixture of four prevalidated siRNAs) or matched negative control (non-target control SMARTpool), both purchased from GE Healthcare Dharmacon (Epsom, UK).

### 4.5. Human Tumor Metastasis PCR Array and qPCR Analysis

Total RNA was extracted from the same HMEC cell populations analyzed for migration (see Figure 5) using PeqGold RNAPure (Peqlab, Erlangen, Germany) and purified according to manufacturer’s instructions. Reverse transcription was done according to cDNA synthesis kit supplied by SA Biosciences. qPCR analysis was performed with the RT^2^ Profiler^TM^ PCR Array “Human Tumor Metastasis” (Cat.# APH-028, SABiosciences/Qiagen, Hilden, Germany) on an I-Cycler (BioRad) using Maxima SYBR Green Mastermix (Thermo Fisher Scientific, Waltham, MA, USA). Data were normalized to the mean expression of 6 housekeeping genes.

### 4.6. Immunoblotting

Confluent cells were washed once with ice-cold PBS and lysed with 1 × PhosphoSafe lysis buffer (Merck Millipore). Cell lysates were sonicated and centrifuged for 10 min at 14,000× *g* and 4 °C following determination of total protein concentration in supernatants using BioRad DC protein assay. Samples containing equal amounts of protein were prepared using 3× SDS sample buffer and 125 mM dithiothreitol (both from New England Biolabs, Ipswich, MA, USA), subjected to gel electrophoresis using BioRad mini-PROTEAN TGX any-kD precast gels and blotted to 0.45 μm PVDF membranes. Membranes were blocked with nonfat dry milk (Carl Roth GmbH, Karlsruhe, Germany) or BSA (Sigma-Aldrich) and incubated with primary antibodies at 4 °C overnight. HRP-linked secondary antibodies and Amersham ECL Prime Detection Reagent (GE Healthcare Dharmacon) were used for detection of proteins on a BioRad ChemiDoc XRS imaging system. RotiFree stripping buffer (Carl Roth GmbH) was used for membrane stripping.

### 4.7. Flow Cytometry Analysis of HMEC

Cell cycle analysis in HMEC was performed as described previously [11]. Briefly, 10^5^ cells were fixed in 70% (*v*/*v*) ice-cold ethanol at 4 °C for 24 h. Thereafter, the fixed cells were stained with CyStain DNA 2 step kit (Partec GmbH, Münster, Germany) and filtered through a 50 μm filter. The samples were then analyzed in a Galaxy flow cytometer (Partec GmbH) using the MultiCycle cell cycle software (Phoenix Flow Systems Inc., San Diego, CA, USA).

### 4.8. Migration and Invasion Assays

The xCELLigence^®^ DP system (ACEA Biosciences, distributed by OLS, Bremen, Germany) was used to measure random migratory activity (chemokinesis) of HMEC, MDA-MB-231 and MDA-MB-435S cells. Cells were seeded in 6-well plates, treated as desired, and then serum-starved (standard growth medium containing 0.5% FCS) for 24 h prior to transferring the cells to the assay. For all assays, RPMI with 1% FBS and a final concentration of TGF-β1 of 5 ng/mL in both the upper and lower chambers of the CIM plates-16 was used. CIM plates-16 were prepared according to the instruction manual and previous descriptions [35]. The underside of the upper chambers of the CIM plate-16 was coated with 30 μL of collagen I (400 μg/mL) and allowed to dry for at least 2 h prior to plate assembly. 40,000 cells were loaded into each well of the upper chamber immediately after addition of TGF-β1 to the cell suspensions. In the invasion assay, the surface of the upper chamber was covered with a monolayer of 5% (*v*/*v*) growth factor-reduced Matrigel (BD Biosciences, diluted 1:40 with basal medium as detailed by Roche in the application notes). Cell indices were measured every 15 min for up to 40 h with the RTCA software (version 1.2, ACEA).

### 4.9. Statistical Analysis

Statistical significance was calculated using the Mann-Whitney u test. Results were considered significant at *p* < 0.05 (*). Higher levels of significance were *p* < 0.01 (**) and *p* < 0.001 (***).

## 5. Conclusions

These data demonstrate that enhanced TGF-β signaling particularly in HMEC is considered anti-oncogenic by suppressing oncogene-induced transformation. However, these effects are associated with a higher migration and invasion potential in senescent HMEC which is accompanied by downregulated Rac1b and upregulated Rac1 expression. Together with additional signals mediated by elevated fibrosis/necroptosis and damage-associated molecular patterns, these events can contribute to aging-related breast cancer development [23].

## Figures and Tables

**Table 1:** Microarray analysis of HMEC P11/P12 vs. HMEC P15/P16 with RT^2^ Profiler PCR Array Human Tumor Metastasis. List of genes with differences in Ct values >1.5 in HMEC P15/P16. Bold indicates induction, italics indicates reduction. Data are the mean of four replicates (two assays, each with two replicates).

HMEC		P11/P12	P15/P16
Gene	Protein		
*APC*	Apc	34.5	**30.8**
*CD44*	CD44/hyaluron receptor	29.3	**26.9**
*CDKN2A*	p16INK4A	32.7	**30.4**
*COL4A2*	collagen 4A2	37.2	**35.2**
*CXCR2*	CXCR2	32.3	**30.1**
*CXCR4*	CXCR4	39	**35.5**
*EPHB2*	Ephrin B2 receptor	28.1	**26**
*ETV4*	ETS variant 4	28.8	**27.2**
*FXYD5*	FXYD domain-containing ion transport regulator 5	36.2	**33.5**
*IL18*	IL18	27	**24**
*KRAS*	Ki-Ras	34.3	**30.8**
*MET*	Hepatocyte growth factor receptor	33.4	**30.9**
*MGAT5*	N-acetylglucosaminyltransferase V	31.8	**28.7**
*MMP2*	MMP-2	31.6	**29.5**
*NME4*	non-metastatic cells 4	27.6	**25.5**
*NR4A3*	nuclear receptor subfamily 4 group A member 3	23.8	**21.8**
*PLAUR*	urokinase-type plasminogen activator receptor	31.2	**28**
*RORB*	retinoid-related orphan receptor β	29.3	**26.7**
*SMAD2*	SMAD2	31	**28.3**
*SMAD4*	SMAD4	33.2	**30.9**
*KISS1*	Kisspeptin-10	35.6	*37*
*KISS1R*	GPR54	35.5	*37*

## Abbreviations

HMEC	Human mammary epithelial cells
MnSOD	Manganese-dependent superoxide dismutase
ERK	Extracellular signal-regulated kinase
TGF-β	Transforming growth factor-β
